# Mismatch Intolerance of 5′-Truncated sgRNAs in CRISPR/Cas9 Enables Efficient Microbial Single-Base Genome Editing

**DOI:** 10.3390/ijms22126457

**Published:** 2021-06-16

**Authors:** Ho Joung Lee, Hyun Ju Kim, Sang Jun Lee

**Affiliations:** Department of Systems Biotechnology and Institute of Microbiomics, Chung-Ang University, Anseong 17546, Korea; hojlee@cau.ac.kr (H.J.L.); yeolmae@cau.ac.kr (H.J.K.)

**Keywords:** single base, microbial genome editing, 5′-truncated sgRNA, Cas9, CRISPR

## Abstract

The CRISPR/Cas9 system has recently emerged as a useful gene-specific editing tool. However, this approach occasionally results in the digestion of both the DNA target and similar DNA sequences due to mismatch tolerance, which remains a significant drawback of current genome editing technologies. However, our study determined that even single-base mismatches between the target DNA and 5′-truncated sgRNAs inhibited target recognition. These results suggest that a 5′-truncated sgRNA/Cas9 complex could be used to negatively select single-base-edited targets in microbial genomes. Moreover, we demonstrated that the 5′-truncated sgRNA method can be used for simple and effective single-base editing, as it enables the modification of individual bases in the DNA target, near and far from the 5′ end of truncated sgRNAs. Further, 5′-truncated sgRNAs also allowed for efficient single-base editing when using an engineered Cas9 nuclease with an expanded protospacer adjacent motif (PAM; 5′-NG), which may enable whole-genome single-base editing.

## 1. Introduction

The CRISPR/Cas9 system consists of a modular single-molecular guide RNA (sgRNA) and a Cas9 protein capable of recognizing DNA target nucleotide sequences and subsequently cleaving the double-stranded target DNA. Therefore, the effectiveness of this technology as a genome-editing tool largely depends on the design of the sgRNAs [1,2]. Unlike CRISPR/Cas9-mediated target cleavage and non-homologous end joining (NHEJ), which may introduce unexpected mutations [3,4], incorporating donor DNA templates such as oligonucleotides, plasmids, and viral vectors allows for more accurate genome editing through homology-directed repair (HDR) [5,6].

However, CRISPR/Cas9 can non-specifically cleave double-strand DNA sequences that are similar to the desired target, which is referred to as an off-target effect [7]. Even if there is a mutation in the protospacer (invading DNA fragments that can be processed to become CRISPR spacers), or a mutation in the guide RNA (gRNA) sequence in CRISPR loci, micro-organisms with CRISPR as an immune mechanism must recognize the target and perform double-strand breaks to survive a phage invasion. Therefore, it is assumed that the CRISPR/Cas system has evolved to allow a certain degree of homology for the target sequence and spacer [8]. In fact, Cas9 has a tolerance of several base mismatches and truncations [9,10]. Several studies have conducted CRISPR/Cas9-mediated microbial genome editing via oligonucleotide-directed mutagenesis [11,12]. In *Escherichia coli*, double-strand breaks of unedited chromosomal DNA can kill the cells because it lacks the NHEJ pathway. Therefore, only edited cells can survive and be obtained, and this is called negative selection. However, the introduction of single-base mutations into microbial genomes by CRISPR/Cas9 is rarely reported, which is likely due to mismatch tolerance.

Given that the sgRNA forms a complex with the Cas9 protein and mediates the specific binding between the target DNA and Cas9 nuclease [13], the target-recognizing sequence of the sgRNA acts as an important factor that determines target specificity and genome editing efficiency [14,15]. However, target cleavage still occurs even if there are several mismatches between the sgRNA and the target DNA [16,17]. Moreover, the 5′-truncated sgRNA/Cas9 complex can also cleave the target DNA despite the reduction in DNA:RNA hybrids [18,19], and even achieve allele-specific genome editing in mammals [20]. In higher eukaryotic genome editing using CRISPR/Cas9, the off-target effect is likely the result of mismatch tolerance, which can generate unwanted mutations in unintended genomic regions [21]. Therefore, an engineered version of the Cas9 protein is often used to avoid double-strand breaks. For example, deactivated Cas9 and base deaminase fusion proteins, referred to as base editors, can induce single-base editing without double-strand breakage [22,23]. A glycosylase base editor was recently developed to introduce transversion mutations, thus widening the potential of base editing [24]. 

Nevertheless, single-base-level genome editing is rarely achieved, even in microbial cells, which is likely due to mismatch tolerance. We recently used CRISPR/Cas9 and Cpf1 to design a mismatched sequence in the sgRNA target recognition sequence (TRS) in advance, to overcome mismatch tolerance, thereby enabling site-specific single-base editing in a bacterial cell with high efficiency [25,26]. Here, we demonstrate a simple and effective single-base microbial genome editing method that can overcome single-base mismatch tolerance using a 5′-truncated sgRNA/Cas9 complex. 

## 2. Results

### 2.1. Mismatch Intolerance between the DNA Target and 5′-Truncated sgRNA

**Figure 1:**
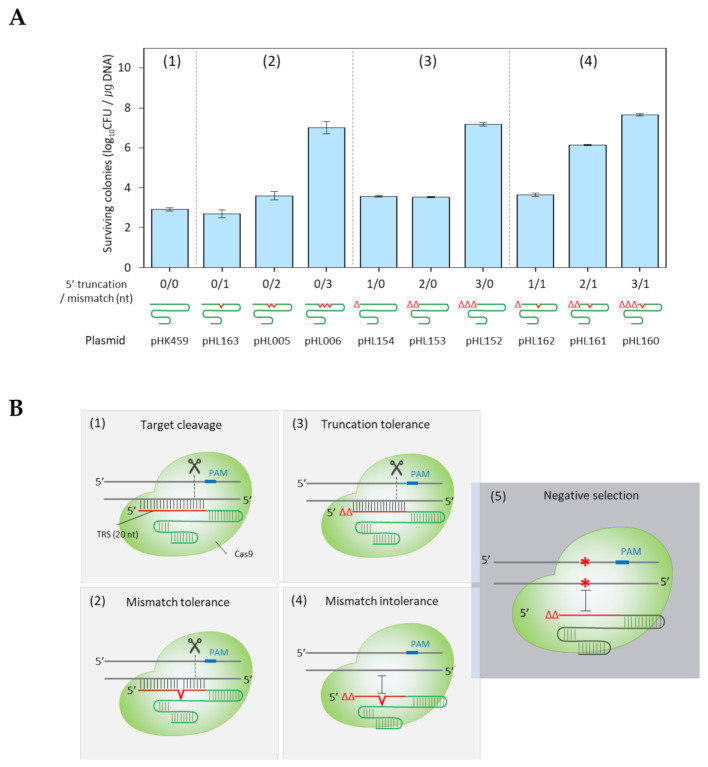
Surviving colonies using 5′-truncated and/or mismatched sgRNA plasmids. (**A**) Number of surviving colonies using sgRNAs carrying the target-matched target recognition sequence (TRS) (1), one to three mismatches (2), 5′- nucleotide truncations (3), and both 5′-nucleotide truncations and single-base mismatches (4). Each bar represents the standard deviation of three independent experiments. (**B**) The target was cleaved by the target-matched (1), -mismatched (2), and 5′-truncated (3) sgRNA/Cas9 complex due to mismatch and truncation tolerance. The 5′-truncated and single-mismatched sgRNA/Cas9 complex cannot cleave the single-base-edited target due to the increased mismatch number (4). Finally, single-base-edited cells could be obtained via negative selection with truncated sgRNA (5). The red lines indicate the TRS.

Although the original TRS of the sgRNA in CRISPR/Cas9 consists of 20 bases, target-mismatched [16,17] or 5′-truncated [18,19] sgRNA/Cas9 complexes can also, reportedly, cleave the DNA target. *E.* *coli* cells in which Cas9 expression had been induced with L-arabinose were transformed with plasmids carrying the sgRNAs with from one to three base mismatches (M) or serially truncated 5′-nucleotides (T). The efficiency of each mismatched or 5′-truncated sgRNA/Cas9 complex, in recognizing and cutting the *galK* target (^498^AGGCTGTAACTGCGGGATCA^517^), was compared by counting the number of surviving colonies on Luria–Bertani (LB) agar containing the appropriate amount of spectinomycin. A decrease in the number of surviving colonies indicates that the CRISPR/Cas9 system operates and cleaves the target DNA, finally causing cell death. Less than 10^4^ CFU/μg sgRNA plasmid DNA were observed when one or two nucleotides were mismatched (T/M = 0/1, and 0/2) or deleted (T/M = 1/0, and 2/0) from the sgRNAs (Figure 1a). However, a large number of colonies survived (>10^7^ CFU/μg) when three nucleotides were mismatched (T/M = 0/3) or truncated (T/M = 3/0) from the sgRNA. These results indicate that mismatch or truncation tolerance for the *galK* target is effective up to two base mismatches (T/M = 0/2) or two nucleotide truncations (T/M = 2/0) in the sgRNAs.

Next, we tested whether sgRNA/Cas9 could cut the *galK* target when the sgRNA had both a 5′-truncation (T) and a single-base mismatch (M) relative to the target. The number of surviving colonies of T/M = 0/1, 1/1, and 3/1 did not change compared to T/M = 0/0, 1/0, and 3/0, respectively. However, the number of surviving colonies increased significantly (10^6^~10^7^ CFU/μg) when T/M = 2/1. These results demonstrate that an additional single mismatch prevents the 5′-truncated sgRNA/Cas9 complex from recognizing and cleaving the DNA target. Based on these results, we hypothesized that this 5′-truncated sgRNA/Cas9 complex could be used to negatively select targets with single-base modifications (Figure 1b).

### 2.2. Negative Selection of Single-Base-Edited Targets by 5′-Truncated sgRNAs

**Figure 2:**
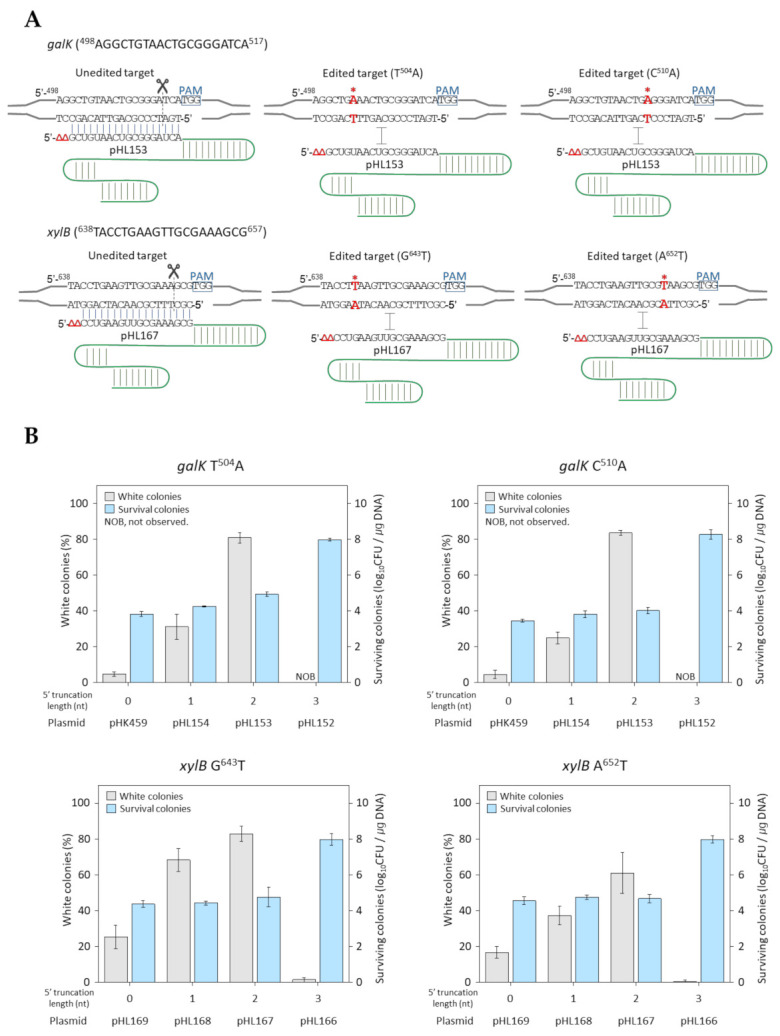
Efficiency of single-base editing by truncated sgRNA/Cas9-mediated negative selection. (**A**) Target sequences and design of truncated sgRNAs for the negative selection of edited targets in *galK* and *xylB*. Asterisk represent single point mutations. (**B**) Efficiency of single-base editing in *galK* and *xylB* target using 5′-untruncated, 1 nucleotide-, 2 nucleotide-, and 3 nucleotide-truncated sgRNAs. Each bar represents the standard deviation of three independent experiments.

Plasmids carrying 5′-truncated sgRNAs and single-base mutagenic oligonucleotides such as *galK*T504A (generating TGA stop codon; causing C168X in GalK protein), *galK*C510A (TGA; C170X), *xylB*G643A (TAA; E215X), and *xylB*A652T (TAA; K218X) were combinatorially electroporated into *E. coli* cells overexpressing Cas9 and lambda Bet proteins. The sequence information of plasmids and mutagenic oligonucleotides are listed in Supplementary Tables S1 and S3, respectively. Two different targets (5′-proximal and 5′-distal) in both the *galK* and *xylB* genes were tested for single-base editing (Figure 2a). If single-point mutations causing premature translational termination are properly introduced into the galactokinase (Gene ID: 945358 (*galK*)) or xylulokinase (Gene ID: 948133 (*xylB*)) genes in the genome, single-base-edited cells cannot ferment D-galactose or D-xylose, therefore white colonies on MacConkey agar plates are produced. The unedited cells can ferment D-galactose or D-xylose, thus red colonies are produced on MacConkey agar plates due to the lowered pH caused by D-galactose or D-xylose fermentation metabolites. Consequently, the ratio of white colonies compared to the total colonies can be used as an indicator of CRISPR/Cas9-mediated single-base editing efficiency. In the case of *galK*T504A single-base editing, less than 5% of white colonies were obtained when untruncated sgRNA was used. However, white colony ratios increased to 31% and 80% when 1 or 2 nucleotides were 5′-truncated, respectively (Figure 2b). Another instance of *galK* 5′-distal single-base editing (*galK*C510A) resulted in 4%, 24%, and 83% when untruncated, 1-nucleotide truncated, and 2-nucleotides-truncated sgRNAs were used, respectively.

In the case of the *xylB* target, 5′-proximal *xylB*G643A and 5′-distal *xylB*A652T were tested using 5′-untruncated, 1-nucleotide-truncated, and 2-nucleotides-truncated sgRNAs. Although 18–25% of white colonies were already obtained using untruncated sgRNAs, the white colony ratios increased significantly as the number of truncated nucleotides (up to two) at the sgRNA 5′ end increased. The increase in the white colony ratio according to the increase in the number of truncated nucleotides of sgRNA was observed in both *galK* and *xylB* gene point mutation experiments. Lastly, few or no white colonies were obtained when the sgRNAs with three truncated 5′-nucleotides were used, and the number of surviving cells, which is the sum of white colonies (edited) and red colonies (not edited), increased to 10^8^ CFU/µg. These findings indicate that the truncation of two nucleotides at the 5′ end of the sgRNAs renders optimal results for negative selection in microbial single-base genome editing.

### 2.3. Editing of Multiple Single-Base Positions Using 5′-Truncated sgRNAs

We next investigated whether sgRNA 5′ truncation could effectively enable the modification of multiple single-base positions in the target sequence, as well as whether each given base could be converted into any of three alternative bases (e.g., A to either C, G, or T). Based on the results described above, the sgRNAs with two 5’ truncations and various mutagenic oligonucleotides were electroporated into *E. coli* cells with highly expressed Cas9 and lambda Bet proteins. There were 30 single-base edits (=10 targets × 3 mutagenic oligonucleotides) in *galK* and 24 single-base edits (=8 targets × 3 mutagenic oligonucleotides) in *xylB*. The efficiency of single-base editing was determined via direct Sanger sequencing of the edited DNA targets of four randomly selected colonies grown on the LB spectinomycin agar plate. The Sanger sequences of edited targets are in Supplementary Figures S2 and S3.

**Figure 3:**
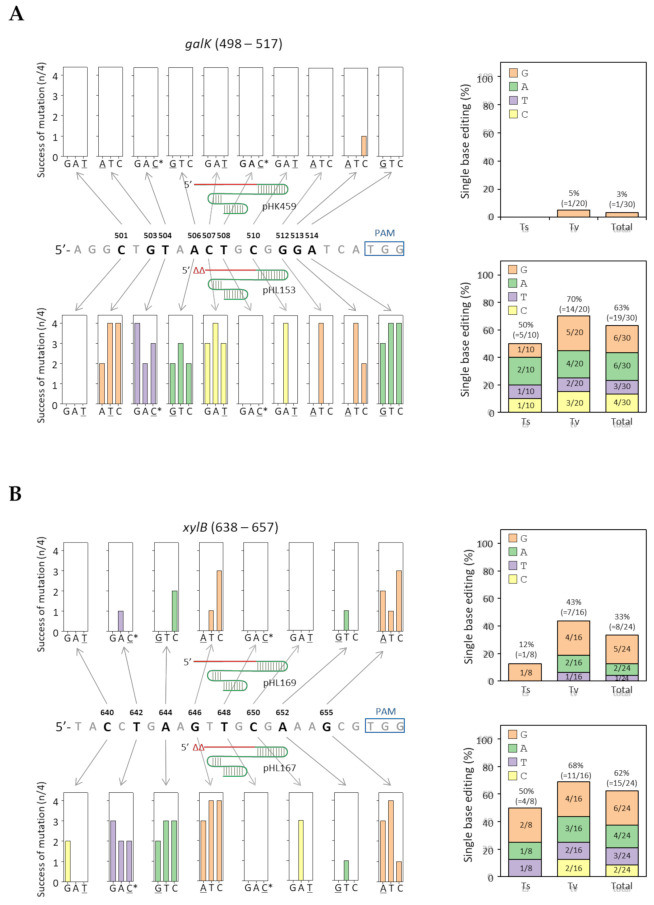
Efficiency of truncated sgRNA-mediated single-base editing in randomly selected colonies. Location of mutation sites and results of single-base editing of various bases in *galK* (**A**) and *xylB* (**B**) targets. Single-base genome editing efficiency divided by mutation types (right). The numbers in the colored boxes and parentheses indicate the frequency and success of single-base genome editing, respectively. * Four single-base edits generate a G:U wobble-base pairing. Underlined bases indicate a transition mutation. Notably, there were twice as many transversion cases than transition alteration cases.

With the exception of one *galK* target (G513C), the editing of 30 single-base alterations at ten different locations failed when 5′-untruncated sgRNA was used (pHK459). In contrast, 19 base alterations out of 30 different cases (19/30 = 63%) were successful when an sgRNA with two 5′ truncated nucleotides was used (pHL153). Regarding ^503^G, ^504^T, ^506^A, ^507^C, and ^514^A in the *galK* target, any given nucleotide could be successfully converted to any of the three other bases (Figure 3a). In the case of ^510^C, ^512^G, and ^513^G certain changes occurred at higher frequencies. In contrast, no alterations were observed in the ^501^C and ^508^T bases among the four randomly selected colonies. Regarding base editing types, transversion alterations (14/20 = 70%) had a slightly higher editing rate than transition changes (5/10 = 50%). 

In the case of the *xylB* target, eight base alterations out of 24 cases were successful using a 5′-untruncated sgRNA (pHL169). When a sgRNA with two 5′ truncations (pHL167) was used, 15 base changes out of 24 cases were successfully obtained with an even higher rate of mutation success (n/4) among the four selected colonies. Transversion base editing (11/16 = 68%) occurred more frequently compared to transition editing (4/8 = 50%) in the *xylB* target (Figure 3b). 

### 2.4. Truncation Tolerance of sgRNA for Cas9-NG with Expanded PAM

**Figure 4:**
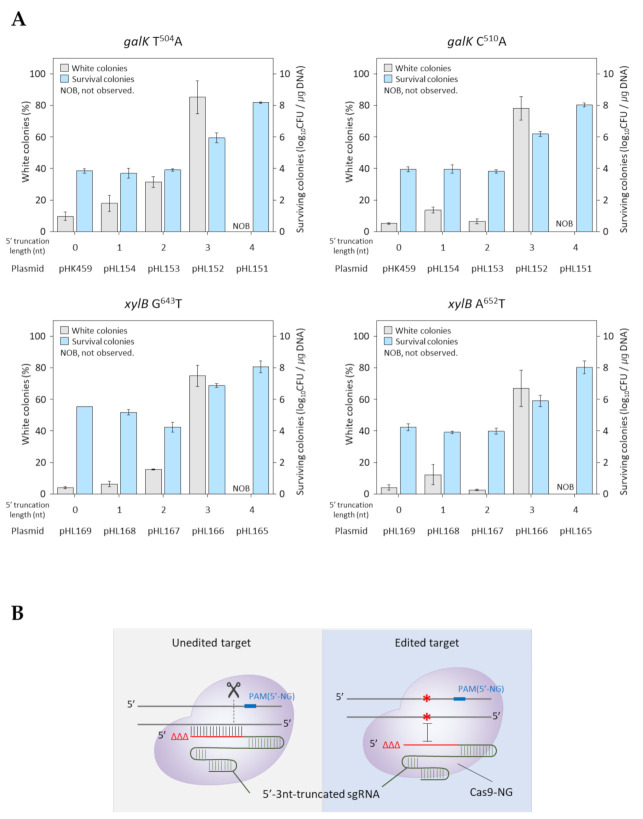
Efficiency of single-base editing with truncated sgRNA/Cas9-NG-mediated negative selection. (**A**) Editing efficiency of the *galK* and *xylB* mutation using 5′-untruncated, 1 nucleotide-, 2 nucleotide-, and 3 nucleotide-truncated sgRNAs. (**B**) 5′-truncation of three nucleotides in the sgRNA is optimum for Cas9-NG-mediated negative selection of single-base edited targets in the microbial genome. Each bar represents the standard deviation of three independent experiments.

Next, we tested the negative selection of the 5′-truncated sgRNA/Cas9-NG complex during oligonucleotide-directed microbial genome editing to expand the target range in the bacterial genome. In the case of the T504A alteration in the *galK* gene, 5′-untruncated, 1 nucleotide-, and 2 nucleotide-truncated sgRNAs resulted in a 9%, 17%, and 31% occurrence of white colonies in the MacConkey D-galactose agar plate, respectively. In contrast, using a 3 nucleotide-truncated sgRNA resulted in an 85% white colony rate with an increased number of surviving cells (~10^6^/µg DNA) (Figure 4a). No white colonies were observed when a 4 nucleotide-truncated sgRNA was used. In the case of C510A in the *galK* gene, 78% of white colonies with many surviving colonies (~10^6^/µg DNA) were similarly obtained using a 3 nucleotide-truncated sgRNA. Similarly, in the cases of G643T and A652T in the *xylB* gene, 3 nucleotide-truncated sgRNAs resulted in white colony rates of 74% and 66%, respectively, in MacConkey D-xylose agar plates. These results indicate that the 5′-truncation of three nucleotides in a sgRNA is required for Cas9-NG-mediated negative selection of single-base edited targets in the microbial genome (Figure 4b).

## 3. Discussion

Off-target effects are a major limitation of current genome-editing methods based on the cleavage of a target gene using CRISPR/Cas9 [7], as Cas9 often cuts the target DNA even without perfect RNA:DNA base pairing between the sgRNA and the DNA target. Therefore, to protect the microbial cells from phage invasion, CRISPR/Cas9 presumably evolved to cut the target DNA sequence even if mutations occurred in foreign genes (e.g., phage or plasmid mutations) or in the crRNA spacer. We also speculated that indel-mediated 5′-truncation of crRNA could also be interpreted as a kind of mismatch (i.e., between the target DNA or the crRNA) that the immune system must handle [8].

According to our findings, target recognition becomes poor when both truncation and mismatch occur at the same time (Figure 1a). CRISPR/Cas9 was used as a negative selection tool for single-base mutations in the microbial genome. When CRISPR/Cas9 negative selection is performed using 5′-truncated sgRNAs, cells with unedited DNA targets in the genome are recognized as targets through truncation tolerance, and are killed through double-strand cutting. Edited cells survive because truncation and mismatch are jointly incorporated, and are therefore not recognized as a target (Figure 1b). 

The results of the 5′-truncation experiments demonstrated that negative selection is not possible in *E. coli* cells with 3 nucleotide-truncated sgRNA in CRISPR/Cas9 (Figure 2b). Ricci et al. reported that more than four mismatches from the 5′-end of the target sterically inhibited the interaction between the target and gRNA, and therefore the conformational activation of Cas9 could not be achieved according to structural simulations [9]. Moreover, based on crystal structure analyses, nucleotides at the 5′-end of gRNA can bind to the RuvC domain in a Cas9 protein via hydrogen bonding and hydrophobic interactions [27]. It is assumed that mismatch can prevent a conformational change in the Cas9 protein to an active state by opening the RNA:DNA hybrid; however, truncation may not play a role as a conformational trigger of the nuclease domain because of the disappearance of the 5′-nucleotides interacting with RuvC. 

One important advantage of the 5′-truncated sgRNA method is that it can be used to edit several bases in the target by using a sgRNA plasmid. Regardless of the editing types (transition/transversion) and editing locations in the target (PAM-distal/proximal), several DNA targets in the *galK* and *xylB* genes could be modified with single-base precision (Figure 3). T504C and T642C transition mutations were obtained in the *galK* and *xylB* genes, respectively, indicating that G:U base pairings can be negatively selected using the 2 nucleotide-truncated sgRNA method. However, we failed to edit T508 and T648 located 10 nucleotides away from the PAM sequence on the *galK* and *xylB* targets among the four randomly selected colonies. Single-base editing was attempted in the *ypdA* and *yjhF* genes to assess whether the location of the mutation site (10 nucleotides from PAM) was not properly edited. As a result, single-base editing was successful at the 10 nucleotide positions in the *ypdA* and *yjhF* targets (Supplementary Figure S1). 

Although it is possible to edit multiple base types and locations in the target using the 5′-truncation method, it is not possible to edit the entire microbial genome due to the limitations of PAM (5′-NGG) in the Cas9 protein. We tested the 5′-truncation method on Cas9-NG nuclease with an expanded PAM (5′-NG) to edit the entire microbial genome (Figure 4a). Unexpectedly, in the case of the Cas9-NG protein, the sgRNA with three 5′ truncations were the most efficient for the negative selection of single-base-edited targets in both the *galK* and *xylB* genes. Additionally, we observed an increased cell survival (10^6^/µg DNA) when 3 nucleotide-truncated sgRNA/Cas9-NG was used for negative selection. This was likely due to the different effects on in vivo nuclease activity compared with those of 2 nucleotide-truncated sgRNA/Cas9. 

Genome editing that changes two or three bases without changing the amino acid codon has been thought to be somewhat adequate. However, synonymous codons induce alterations in the translation elongation rate due to ribosome stalling during protein synthesis. This allows for a protein with altered conformation or specificity for the substrate to be synthesized in the cell [28,29]. In contrast, the 5′-truncation method can be used to accurately edit not only codons, but also regulatory genes in the genomes of microbial strains in the desired direction, or repair them to the desired sequence. Therefore, this approach can be used to engineer customized and precise recombinant microbial strains.

## 4. Materials and Methods

### 4.1. Strains and Culture Conditions

The *E. coli* strains used in this study are listed in Supplementary Table S1. Each strain was cultured in LB broth at 30 or 37 °C depending on their plasmid *ori* sequence. *E. coli* DH5α was used as a cloning host for the construction of the sgRNA plasmids. *E. coli* MG1655 was constructed to harbor the *cas9* [26] or *cas9-NG* gene in their chromosomes. The construction of an *E. coli* strain carrying the *cas9-NG* gene in the chromosome is described below. When necessary, 50, 25, and 75 μg mL^−1^ of ampicillin, kanamycin, and spectinomycin were added to the culture medium.

### 4.2. Genomic Integration of cas9-NG

To integrate the *cas9-NG*-KmR cassette in the *E. coli* genome, we inserted the *cas9-NG*-KmR cassette using a previously described recombineering technique [30]. The *cas9* gene was codon-optimized using the Codon Optimization Tool (https://sg.idtdna.com/pages/tools/codon-optimization-tool, accessed on 1 June 2021) to carry seven point mutations (encoding R1335V, L1111R, D1135V, G1218R, E1219F, A1322R, and T1337R) and to be chemically synthesized (Integrated DNA Technologies, Coralville, IA, USA). Briefly, the *cas9-NG*-KmR cassette was fused using an overlap polymerase chain reaction (PCR) with 5′ fragments of the *cas9* gene (3 kb), chemically synthesized 3′ fragments of the *cas9-NG* gene (837 bp), and a kanamycin resistance marker. The purified PCR fragments were electroporated into L-arabinose-induced *E. coli* MG1655 harboring pKD46 to make *E. coli* HK1159 (Δ*araBAD*::*P_BAD_*-*cas9-NG*-KmR). Subsequently, plasmid pKD46 was cured out of the strain by incubation at 42 °C, and the successful incorporation of the *cas9-NG* gene was confirmed via Sanger sequencing. 

### 4.3. Plasmid Construction

Supplementary Table S1 summarizes the 5′-truncated sgRNA plasmids used for single-base editing. The sgRNA expression plasmid vectors were constructed by targeting the *galK* and *xylB* in *E. coli* for single-base genome editing. Two DNA fragments containing a spectinomycin resistance gene or the sgRNA gene were amplified with primers, listed in Supplementary Table S2, using pHK459 [31] and pHL003 [26] as a template. All of the sgRNA plasmids were generated via Gibson assembly in the same direction.

### 4.4. Base Editing in Galk and xylB

pHK463, which expresses λ red Bet protein, was used to aid ssDNA mediated recombination [26]. *E. coli* HK1059 and HK1159 harboring pHK463 were grown in ampicillin-containing (50 µg mL^−1^) LB medium at 30 °C. The L-arabinose was added when OD_600_ reached 0.4 to overexpress Cas9 nuclease and λ red Bet protein, which facilitates oligonucleotide-directed mutagenesis [32]. After 3 h, the cells were washed twice with 10% glycerol, aliquoted, and stored at −80 °C for subsequent electroporation of mutagenic oligonucleotides and sgRNA plasmids. Mutagenic oligonucleotides (Supplementary Table S3) were designed to introduce single-base editing (T504A or C510A in *galK*, G643T or A652T in *xylB*). Each introduced a premature stop codon in *galK* and *xylB*. For the negative selection of mutant cells harboring single-base substitutions, 5′-truncated sgRNA plasmids were designed.

Each mutagenic oligonucleotide (100 pmoles) with sgRNA plasmids (200 ng) was electroporated in *E. coli* HK1059 and HK1159 cells harboring pHK463 to introduce single-base edits, as well as for CRISPR/Cas9-mediated negative selection. Electroporation was performed at 25 μF, 200 Ω, and 1.8 kV using a 0.1 cm electroporation cuvette. Afterward, the cells were immediately transferred to 950 mL SOC and incubated at 37 °C and 180 rpm for 1 h for recovery before being spread on MacConkey agar containing D-galactose (0.5%, CAS No. 59-23-4) or D-xylose (0.5%, CAS No. 58-86-6) and spectinomycin (75 μg mL^−1^). The cells were then incubated for 16 h at 37 °C. The genome editing efficiencies of oligo-directed *galK* and *xylB* mutagenesis followed by CRISPR/Cas9 negative selection were calculated by counting the white and red colonies expressing D-galactose- or D-xylose-fermenting phenotypes. Cell survival was also calculated by counting the total colonies on MacConkey (with D-galactose or D-xylose), or LB-agar-containing spectinomycin, to confirm whether target digestion by the CRISPR/Cas9 system was efficient.

### 4.5. Multiple Single-Base Genome Edits

Ten targets in *galK* and eight targets in *xylB* were selected to confirm editing efficiency depending on the mutation site within the N_20_ target sequence. The single-base mutagenic oligonucleotides for each target are listed in Supplementary Table S3. The sgRNA plasmids for these targets were the same as those used for base editing in *galK* and *xylB*. For position-based single-base editing experiments, each single-base mutagenic oligonucleotide (100 pmoles) and the corresponding sgRNA plasmids (200 ng), were electroporated into L-arabinose-induced HK1059 cells harboring pHK463. Electroporated samples were spread on LB agar plates containing spectinomycin (75 μg mL^−1^). After 16 h at 37 °C, four colonies (per electroporation) were randomly selected, and Sanger sequencing was carried out to confirm the desired base editing in the *galK* and *xylB* targets using the primers listed in Supplementary Table S3.

## Data Availability

The data that support the findings of this study are available from the corresponding author upon reasonable request.

## Supplementary Materials

The following are available online at https://www.mdpi.com/article/10.3390/ijms22126457/s1.
