# Supplementary material for: Mismatch Intolerance of 5′-Truncated sgRNAs in CRISPR/Cas9 Enables Efficient Microbial Single-Base Genome Editing
Source: Int J Mol Sci. 2021 Jun 16;22(12):6457. doi: 10.3390/ijms22126457 (PMC8235755; doi:10.3390/ijms22126457)
Supplement: Supplementary file 1 [file ijms-22-06457-s001.zip › 20210612_Supplementary_Figures.pdf]

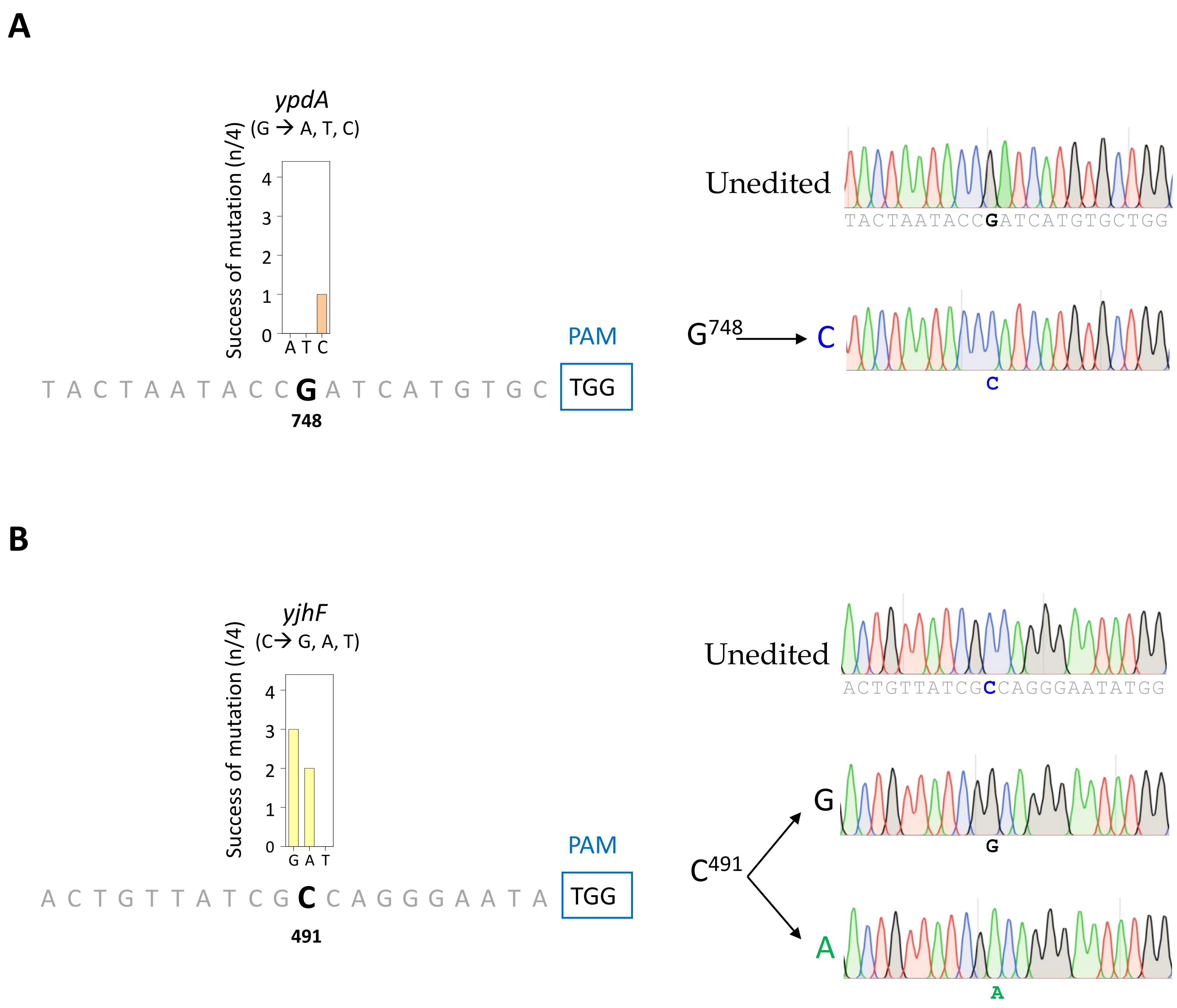

Supplementary Figure S1. Efficiency of truncated sgRNA-mediated single-base editing in a nucleotide located 10 nucleotides away from the PAM sequence. Location of mutation sites and results of single-base editing in *ypdA* (**A**) and *yjhF* (**B**) targets. Sanger sequences of edited targets were provided (right).

Sanger sequences : *galK*(498-517)

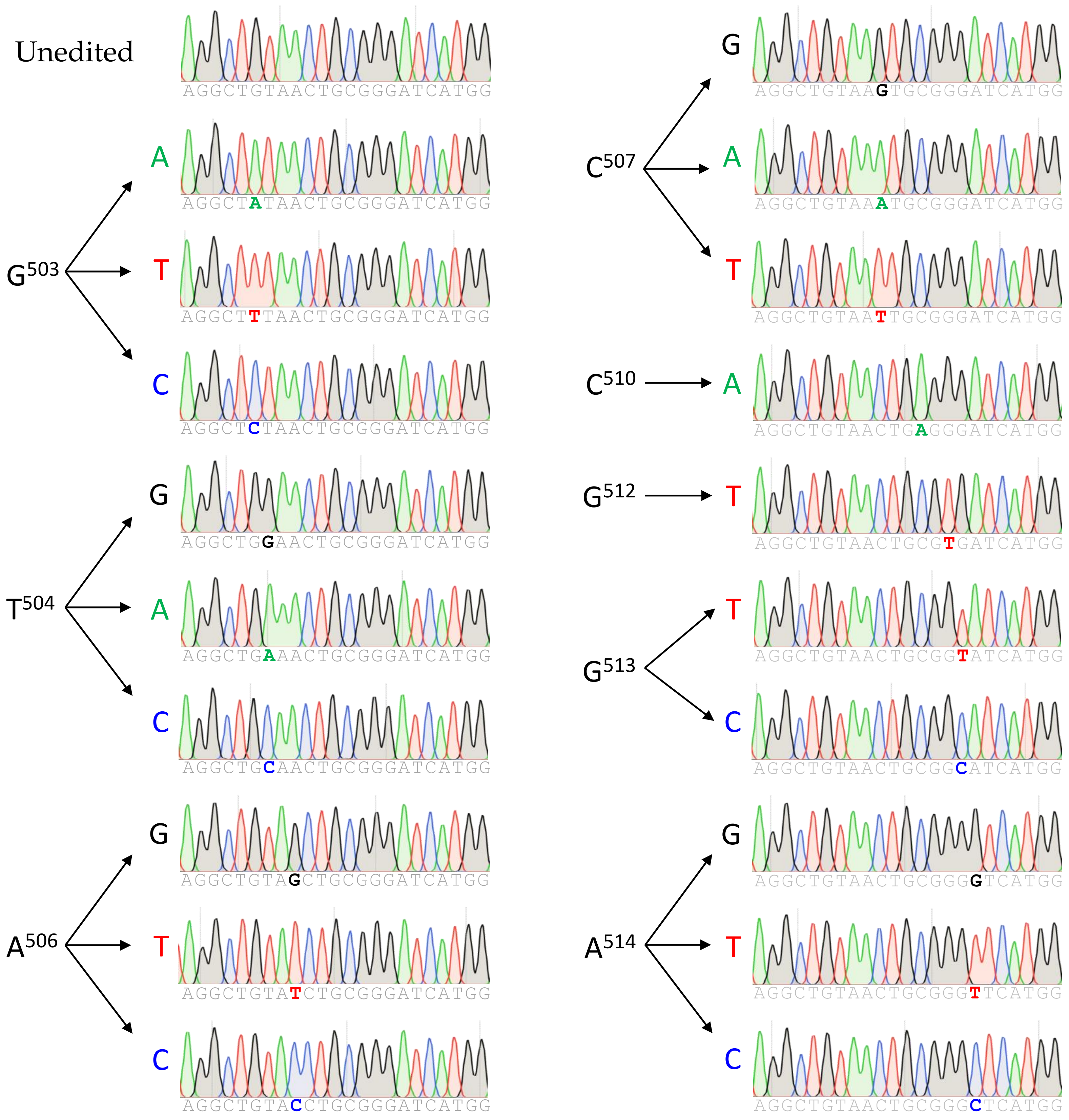

Supplementary Figure S2. Location of mutation sites and Sanger sequencing results of truncated sgRNA-mediated single-base editing of various bases in *galK* 498 – 517 targets.

Sanger sequences : *xylB*(638-657)

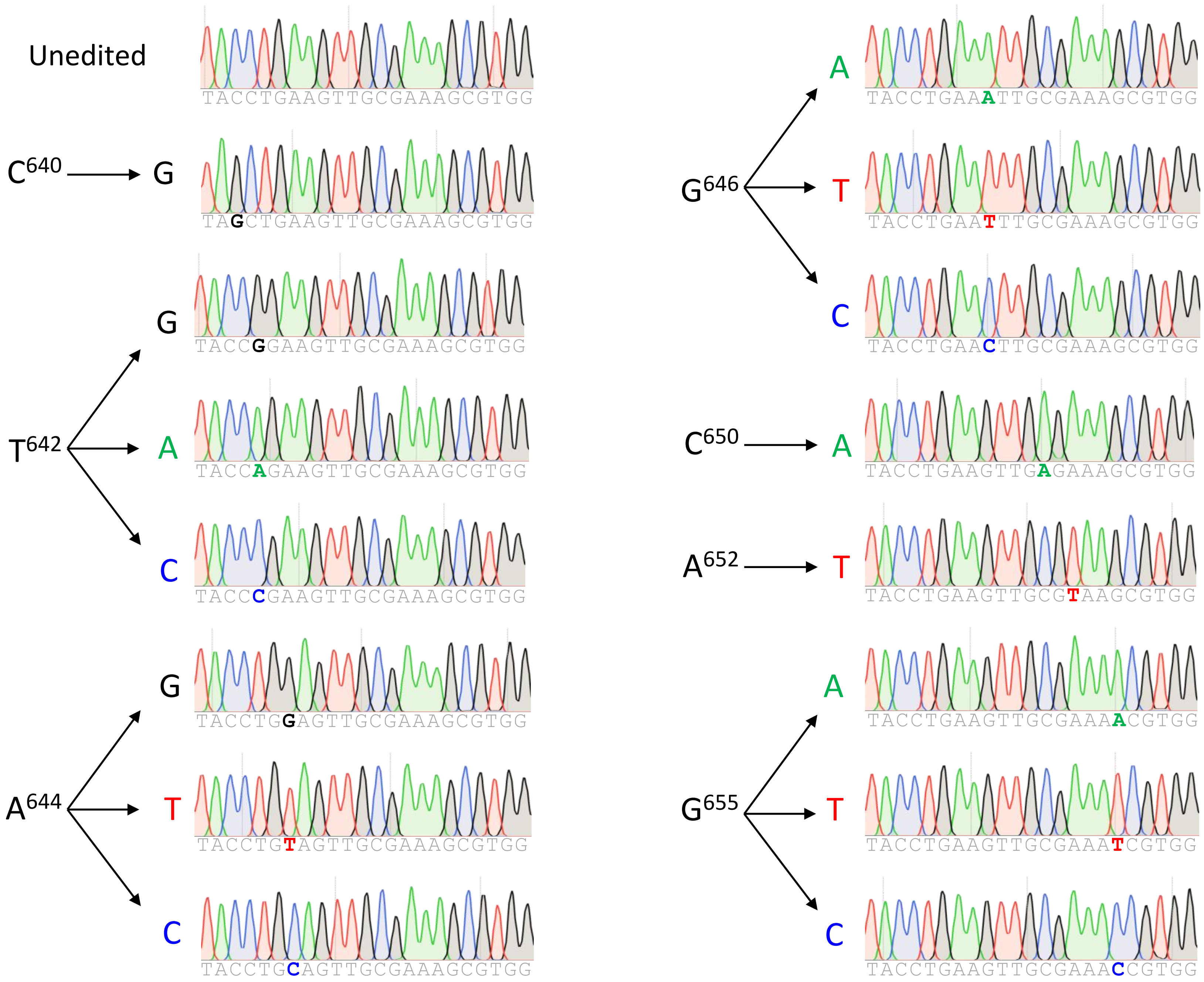

Supplementary Figure S3. Location of mutation sites and Sanger sequencing results of truncated sgRNA-mediated single-base editing of various bases in *xylB* 638 – 657 targets.
